# Points of Interest in a Twenty Years’ Practice

**Published:** 1903-09

**Authors:** Ned A. Stanley

**Affiliations:** New Bedford, Mass.


### POINTS OF INTEREST IN A TWENTY YEARS' PRACTICE.[1]

BY NED A. STANLEY, D.M.D., NEW BEDFORD, MASS.

Mr. President and Brother Alumni,—The subject upon which I am to speak for a few minutes was selected by the committee, of which I believe I am a member, but was not even consulted in the matter. The request, or rather demand, was for a fifteen-minute paper on "Points of Interest in a Twenty Years' Practice."

I am not sure from the subject just what the committee expect, but take it that a few practical points or suggestions from my eighteen years' practice, to be exact, would be somewhat in the line they may have had in mind; but if you do not get anything worth listening to, just blame the committee,—*i.e.*, the other two members.

I think it was Huxley who said, after he had given his first

---

[1] Read before the Harvard Dental Alumni Association, Boston, Mass., Monday, June 22, 1903.

lecture, supposed to occupy one hour, that in half an hour's time he had told everything he knew, and had done nothing but repeat himself ever since. Reasoning from analogy, I should exhaust myself in seven and one-half minutes, and never expect to be called upon again.

However, the subject is so elastic and the length of tether, alas! so generous, that even for one who lacks the power of concentration he cannot fail to carelessly compile a few desultory remarks.

I feel like trying to say something that may be of practical aid to the young practitioner and those about to engage in practice, and yet I shrink somewhat from the undertaking, knowing that the best of instruction and advice has been received by every alumnus, and from those more competent to give.

Happy is the man who has a profession in these unsettled and uncertain days in the business world. A man's personal skill is his own, and nothing but death or accident should deprive him from exercising it. In choosing dentistry for your life-work, bear in mind that you have selected one of the great professions, as well as one of the most exacting, in which it should be your aim to become representative men. I think the feeling often comes in the experience of young professional men that they have chosen unwisely and might have done better at something else. Do not let such thoughts affect you, for they will pass away, and you will come to see, and be in the right relation to your profession.

Newton said he was only picking up an occasional pebble on the shore with the vast expanse of the sea beyond. A touch of agnosticism, which simply means I do not know, is a good thing for all of us, for things in this world move rapidly and without intermission, and the dental profession is one of them.

The possibilities it embraces are bringing men of broad and liberal education to its fold every day. To be a representative man in such a profession means much to any practitioner.

One of the first things in which the young dentist can show his good taste is his office appointments. Upon entering your office the first thing the eye will observe is how it is furnished. Here is where by showing good taste you can, unconsciously, favorably impress the would-be patient before seeing him. Especially is this true in the selection of pictures.

When your professional advice is sought, let one of the first given be, how very essential to the proper care of the mouth and teeth is cleanliness, and never shrink from or shirk this feature of your work, which is, I am afraid, too often the case.

Habits formed early become more readily a part of one's self than those acquired later in life. In beginning practice it should be every man's study how to save physical and nervous force. The man with a large pressing practice is kept at high tension, and every means should be employed enabling us to do our work as easily as possible.

One of the greatest of these is the operating-stool. You can accustom yourself to do nearly everything in an easy, restful position to yourself. For ten years and upward I gave little or no thought to the matter, but there came a time when to stand all day was a different story; and not until I visited Dr. Gillett's office did I appreciate the extent of the use of the operating-stool, everything conforming to the height of a stool perfectly comfortable to sit upon, and everything within easy reaching distance.

I strongly urge this upon your consideration. Do not wait ten years; be wiser than I was and adopt its use at the start. School yourself to take things easily and with as little worry as possible. This advice I can give, but never could follow very well. We know the reason the earth is not hit by travelling meteors is because when they come in contact with the atmosphere the friction generates sufficient heat to destroy them.

Now, some people have the faculty of surrounding themselves with an atmosphere which acts like a shield against troublesome and annoying things, while others less fortunate are being continually pelted. Everything disagreeable seems to strike them in huge lumps, and with the close of each day much good nervous energy has been wasted which might have been put to better use.

As in the days of Hamilton and Jefferson, when to bring order out of chaos it was necessary to have such powerful characters diametrically opposed to each other, so in a lesser sense we have the radical and the conservative practitioner. By all means be guided by the latter.

It has been my opportunity to see much of the work done by one of the most conscientious practitioners and teachers of a generation since, but an extremist of the worst type. New theories were adopted and put into practice with the most disastrous results. Many years ago it was Arthur's separation, the ruthless sacrifice of the first molar by some operators. The adoption of methods and theories which seem plausible but are unsound is sure to breed mischief. As Shylock says to the Christian, " The villany you teach me I will execute, and it shall go hard but I will better the instruction." We can aid and often improve the conditions under which nature does her work, but the man who takes it upon himself to improve upon her general plan is presuming too much.

In recent times, and from which less disastrous results are likely to follow, we have extension for prevention, which I believe in, but hardly to the extent of some of its exponents. The individual case in point and the sound discriminating judgment of the operator must determine the extent of the cutting necessary. Good judgment is the most essential quality demanded by the dentist, for we must decide problems that ten, fifteen, twenty years later reflect upon that judgment. Then, second only, is the marvellously skilled hand with which to carry out what that judgment tells us.

And we all will meet with failures quite often enough. If it were not for our failures progress would be of uncertain growth. Holmes says the man of " facts" lives in a one-story house. Our profession calls for, nay, demands, a resourceful and creative mind, and if we are lacking in this we cannot succeed by doing things as some one tells us.

In the large percentage of cases greater skill is required than in surgery. To be sure, we do not share the responsibility of the surgeon, nor is nature the great healer with us as with him. The responsibility of most of our operations rests with us.

In orthodontia both judgment and skill of a high order are required; and most gratifying it is to know that the students of this school are being taught in this most important branch. Something not even dreamed of in our days of school work. It should form a very important feature in every man's practice. Aside from appearances we all know that teeth in normal position have a great advantage over those in malocclusion.

One other point I wish to touch upon briefly, for in a paper of this nature I should not think of going into technique or detail, and that is, attend society meetings. There is where we can be of help to each other. Unless a man takes interest enough in his profession to meet his professional brother he is going to vegetate; the circle in which he travels will be a small one. We must progress till the end.

Many years ago we had as guest at one of our alumni dinners the late Bishop Brooks. In the course of his remarks, I remember, he spoke of the usefulness and help we were to each other, that the service we rendered to others enabled them to do their work better and with greater comfort.

To-day the torch is in our hands to carry, and let those who follow find that the wave of progress has left its mark higher than before. Let each one of us so work and live that the community shall be the better for our having lived and labored therein.

